# A Case of Rupture of the Womb Occurring during Labor, and Followed by Recovery

**Published:** 1848-02

**Authors:** 


5. *A case of Rupture of the Womb occurring during labor, and followed by recovery.* By Dr. PRASSART.

Cases of recovery after rupture of the womb are of such rare occurrence, that we are desirous of recording the leading features of this one, the subject of which had to contend alike with her formidable accident and the neglect of her attendant. A woman, æt. 37, of muscular make and very choleric temper, had been in labour for six or 8 hours, on 1st of February, when, at about 4 p. m., on getting on the bed she was seized with a tremendous pain, contemporaneously with which the waters were discharged, and a loud cracking sound was heard. She complained of terrible suffering at the umbilical region, and grasped this with both her hands. Labour-pain ceased, and she became ghastly and cold, so that her friends believed her in the act of dying, and had the religious sacraments administered. After a long period, however, they determined to call in advice, and about six hours after the accident the author saw her. He found her suffering from the extremest prostration and intense tenderness of the belly, through the parietes of which the parts of the child were plainly felt. He easily delivered her of the dead child by means of the forceps, a large discharge of blood following. He endeavoured to ascertain the size and position of the aperture, but could only discover that his hand at once passed into the cavity of the abdomen, whence he removed the placenta, and that large coils of intestine passed into the uterus, the great pain induced forcing him to desist. The woman, after this, seemed almost lifeless, and the author informed her friends that she could not live the night. With true Germanic phlegm, he seems to have taken no pains to ascertain whether his prediction was verified, and in *four days* after was much surprised at being again called to visit the patient. He now found severe inflammatory action of the womb and abdomen set up, accompanied by great prostration of strength. We need not follow the case through its remaining details, presenting, as it does, but another example of the occasional wonderful power which Nature employs in coping with the direst extremities of disease. The author, from his unfrequent visits (alternate days) and the nugatory character of his treatment, may be considered as having delivered it over into her hands, the result being, that in four weeks the woman was enabled to leave her bed. She continued for some time after her recovery to be tormented with occasional severe pains; but in the course of the following year natural menstruation was re-established.—*Casper's Wochenschrift*, No. xli, 1847. *Ibid.*

